# Dissecting the multi-omics landscape of TEAD1 in hepatocellular carcinoma: cycle regulation and metastatic potential

**DOI:** 10.3389/fimmu.2025.1567969

**Published:** 2025-06-05

**Authors:** Ruiping Huai, Canquan Mao, Lili Xiong

**Affiliations:** ^1^ Southwest Jiaotong University, School of Chemistry, Chengdu, China; ^2^ Southwest Jiaotong University, School of Life Science and Engineering, Chengdu, China; ^3^ Southwest Jiaotong University, School of Materials Science and Engineering, Chengdu, China

**Keywords:** TEAD1, biomarker, LIHC, single-cell, cell cycle, EMT

## Abstract

**Background:**

The effects exerted by the TEA domain transcription factor family genes on tumorigenesis in various cancers have been extensively investigated. Nevertheless, the potential role of TEAD1 in cancer-related epigenetic alterations, immunological characteristics, and prognosis remains ambiguous. This study aims to clarify the function and potential mechanisms of action of TEAD1 in cancer.

**Methods:**

We assessed pan-cancer expression, methylation, and mutation profiles of TEAD1 to determine its prognostic significance in clinical settings. Furthermore, we analyzed the pan-cancer immunological landscape of TEAD1, with a particular focus on liver hepatocellular carcinoma (LIHC), using correlation analysis. We also performed a subtype-specific analysis of TEAD1 in LIHC to identify its expression patterns, immunological traits, and constructed a prognostic model based on disulfidptosis-related genes. Lastly, we assessed the impact of TEAD1 knockdown on LIHC cell lines HepG2 and Huh-7 by using *in vitro* experiments.

**Results:**

Our findings suggest that TEAD1 is differentially expressed across various cancer types and can act as an independent prognostic factor for multiple cancers. Moreover, we observed that epigenetic changes involving TEAD1 are highly heterogeneous among several cancers; abnormal methylation and copy number variations were associated with a poor prognosis in multiple malignancies, especially in LIHC. Immunoassays demonstrated a significant association between TEAD1 and numerous immune checkpoints in LIHC. Additionally, cellular experiments revealed that knocking down TEAD1 reduced the proliferation, migration, and invasion capabilities of LIHC cells.

**Conclusions:**

The results of this study imply that TEAD1 may serve as a promising prognostic biomarker for tumors and an immunotherapy target, while playing a crucial role in the proliferation, migration, and invasion processes within LIHC.

## 1 Introduction

Cancer continues to exert a substantial global burden, with increasing prevalence and impact across diverse populations. The disease’s escalating incidence and the profound effects on various communities underscore the urgency of intensified research and intervention efforts. Liver hepatocellular carcinoma (LIHC) is the third leading cause of cancer-related mortality and the sixth most frequently diagnosed cancer worldwide, with approximately 906,000 new cases and 830,000 deaths reported in 2020 (1). As the most prevalent primary liver malignancy, LIHC accounts for approximately 90% of all liver cancer cases. Despite advancements in treatment strategies, the majority of LIHC patients are diagnosed at advanced stages, resulting in a five-year survival rate of less than 20% (2). There is an urgent need for a deeper understanding of LIHC pathogenesis and the identification of novel biomarkers.

The TEA domain family of transcription factors is highly conserved and ubiquitously expressed across mammalian tissues, with the four TEA domain genes exhibiting distinct tissue-specific expression patterns (3–6). TEA domain transcription factor 1 (TEAD1), the first member identified within this family, has been implicated in various cancers due to its deregulation (7). knockdown of TEAD1 has been shown to suppress cell proliferation in gastric cancer (8), conversely its overexpression enhances cell proliferation, migration and invasion in pancreatic cancer (9). Similarly, activation of the TEAD1 signaling pathway promotes malignant phenotypes in gastric cancer cells (10). Understanding the complex mechanisms by which TEAD1 contributes to cancer pathogenesis is crucial and holds significant promise for the developing of targeted and personalized therapeutic strategies.

In this study, we conducted a comprehensive analysis of TEAD1’s pan-cancer expression levels, prognostic significance, epigenetic alterations, and immune landscape. We specifically investigated the immunological characteristics and associated with TEAD1 and established a prognostic model for LIHC based on disulfidptosis-related genes. Our findings were validated through *in vitro* experimentation and may provide valuable insights for future research on TEAD1.

## 2 Materials and methods

### 2.1 Datasets acquisition

mRNA expression profiles of normal tissues were obtained from the Genotype-Tissue Expression (GTEx) database (https://www.gtexportal.org/home/) and the Human Protein Atlas (HPA) database (https://www.proteinatlas.org/). Gene expression data for cancer cell lines were retrieved from the HPA database. Copy number variations (CNV), DNA methylation (Methylation450K) data, and TPM format RNAseq data from The Cancer Genome Atlas (TCGA) and GTEx, uniformly processed by the Toil pipeline (11), along with clinical features for 33 cancer types, were sourced from the UCSC XENA platform (https://xenabrowser.net/datapages/). TEAD1 protein expression profiles were extracted from the Clinical Proteomic Tumor Analysis Consortium (CPTAC) database to assess protein expression levels in cancer. To validate the differential expression of TEAD1 across cancers, six datasets (GSE93601, GSE16011, GSE6344, GSE36376, GSE19804, and GSE39791) were sourced from the Gene Expression Omnibus (GEO, https://www.ncbi.nlm.nih.gov/geo/) database, and the validation dataset E-MEXP-1327 for prostate adenocarcinoma (PRAD) was derived from the Affymetrix GeneChip Human Genome HG-U133A platform. Pan-cancer immune cell infiltration data were procured from Tumor Immune Estimation Resource 2.0 (TIMER2.0, http://timer.cistrome.org/). The liver cancer dataset, LIRI-JP, was accessed from the International Cancer Genome Consortium (ICGC, https://dcc.icgc.org/). Single-cell data were obtained from the Tumor Immune Single-cell Hub 2 database(https://tisch.comp-genomics.org/). Finally, information about the spatial transcriptome datasets is provided in **Supplementary Table 1**.

### 2.2 Pan-cancer differential expression, clinical prognostic, and epigenetic analysis of TEAD1

Using HPA and GTEx data, we analyzed the expression level of TEAD1 in normal human tissues and cancer cell lines. Based on TCGA pan-cancer expression profile data, we evaluated the expression of TEAD1 in 33 different cancer types. In addition, the differential expression of TEAD1 was validated based on additional datasets. Using the Clinical module of the TISIDB database, we explored the correlation between TEAD1 and pan-cancer clinical stage. Pan-cancer clinical survival information includes overall survival (OS), progression-free interval (PFI), disease-free interval (DFI), and disease-specific survival (DSS). We grouped all patients into 33 cancer types according to the median expression level of TEAD1 mRNA, and all patients were divided into the TEAD1 high expression group and the TEAD1 low expression group. R packages “survival” and “survminer” were used to perform COX analysis. In addition, we evaluated the CNV and methylation level of TEAD1 in pan-cancer, as well as the association with mRNA expression and clinical prognosis.

### 2.3 Immune-related analysis

The R package ESTIMATE (12) was used to calculate the StromalScore, ImmuneScore, ESTIMATEScore, and TumorPurity of tumor tissues, and the correlation between TEAD1 and different scores was evaluated. The correlation between TEAD1 and immune cell infiltration was evaluated using xCell, ssGSEA, and CIBERSORT algorithms (13–15). In addition, we obtained the information of 122 immune regulators collected by Charoentong et al., including MHC, receptors, chemokines, and immunostimulants (16), and calculated the Pearson correlation between TEAD1 and pan-cancer immune regulators. In addition, we used the TIP (tracking tumor immunophenotype) database (17) to evaluate the anti-cancer immune status at seven different stages of the tumor-immunity cycle: release of cancer cell antigens (step 1), cancer antigen presentation (step 2), priming and activation (step 3), trafficking of immune cells to tumors (step 4), infiltration of immune cells into tumors (step 5), T cell recognition of cancer cells (step 6), and killing of cancer cells (step 7). The Cancer Immunome Database (TCIA) (16) was used to evaluate the relationship between TEAD1 and immunotherapy.

### 2.4 Single-cell and spatial transcriptomic analysis

We downloaded the LIHC single-cell dataset GSE146115 from the TISCH2 (18) database and used the uniform manifold approximation and projection (UMAP) technique to visualize the high-dimensional data into a two-dimensional heatmap, and visualized the expression data of the TEAD1 gene. The Kruskal-Wallis rank sum test was used to evaluate the expression difference of the TEAD1 gene in different cell types. All cells were divided into positive/negative expression groups according to whether the TEAD1 gene was expressed, and the proportion of each cell type in the positive/negative expression group was calculated respectively. The AUCell package was used to evaluate the scores of immune, metabolic, signaling pathways, proliferation, cell death, and mitochondrial-related biological pathways. The limma package was used to compare the differences in scores between the TEAD1 expression positive and negative groups. Based on previous research methods, we processed the LIHC spatial transcriptome data. The Cottrazm package was used to deconvolute different cell components (19). The cell type with the highest content in each microregion was calculated, and the SpatialDimPlot function in the Seurat package was used to visualize the maximum value of the cell component in each microregion and the expression landscape of the TEAD1 gene in each microregion. Spearman correlation analysis was used to calculate the correlation between cell content and cell content in all spots, as well as the correlation between cell content and gene expression, and the linkET package was used for visualization.

### 2.5 Functional enrichment analysis

According to the median expression value of TEAD1, LIHC patients were divided into two groups, namely, the TEAD1 high expression group and the TEAD1 low expression group. The limma package was used to perform differential analysis. Genes with Fold change (FC) greater than 2 and p-value less than 0.05 were considered to have significant differences. Volcano plots were drawn for visualization. The clusterProfiler package completed Gene Ontology (GO) and Kyoto Encyclopedia of Genes and Genomes (KEGG) enrichment analysis. In addition, all genes were sorted according to log2FC, and the clusterProfiler package performed gene set enrichment analysis based on GO-Biological Process (BP) gene set, GO-Molecular Function (MF) gene set, GO-Cellular Component (CC) gene set, reactome gene set, and wikipathways gene set, calculated the gene set enrichment score ES, and performed significance tests and multiple hypothesis tests on the ES values of the gene sets. The top 5 pathways that were significantly enriched in the high/low expression groups were selected for visualization. The z-score parameter in the R package GSVA was used to calculate the gene set and obtain the combined z-score score. We used the scale function to define the gene set score and calculated the Pearson correlation between TEAD1 and each gene set score.

### 2.6 Construction of a prognostic model based on disulfidptosis-related genes

Based on the study of Xu et al. (20), we collected 24 disulfidptosis-related genes. We also performed correlation analysis with TEAD1 to obtain hub genes related to disulfidptosis. Then, we used the lasso-cox regression method to reduce the dimension and build a prognostic model. The specific steps were as follows: the TPM format expression spectrum of TCGA-LIHC was normalized by log2(TPM+1), and samples with RNAseq data and clinical information were retained. The lasso algorithm in the R package “glmnet” was used for feature selection, and 10-fold cross-validation was used. The R package “survival” was combined with multivariate Cox regression analysis to build a prognostic model. Iterative analysis was performed through the step function to select the optimal model. Log-rank was used to test the KM survival analysis to compare the survival differences between the above two or more groups, and timeROC analysis was performed to discriminate the accuracy of the prediction model. Univariate and multivariate Cox analysis was used to determine the potential of risk factors as independent prognostic factors.

### 2.7 Cell culture and transfection

HepG2 and Huh-7 cells were purchased from Shanghai Cell Bank Library of the Chinese Academy of Sciences (Shanghai, China) and incubated with Dulbecco's Modified Eagle Medium (DMEM) (HyClone) with 10% fetal bovine serum (Biological Industries, ISRAEL), 100 U/ml penicillin, and 100 μg/ml streptomycin solution (HyClone) at 37 °C in 5% CO2. Two siRNAs specific targeting TEAD1 and a scramble negative control siRNA were designed and synthesized by GenePharma Company (Shanghai, China). These siRNAs were transfected into HepG2 or Huh-7 cells using the Lipofectamine 3000 Reagent (Invitrogen, California, USA) in accordance with the manufacturer's instructions. The experiment was conducted in triplicate. The sequences of siRNA1 sense(5'-3'): CCACUGCCAUUCAUAACAATT, antisense(5'-3'): UUGUUAUGAAUGGCAGUGGTT. The sequences of siRNA2 sense(5'-3'): CAUGGCCUGUGUGUUUGAATT, antisense(5'-3'): UUCAAACACACAGGCCAUGTT.

### 2.8 RNA extraction and quantitative real-time PCR

Total RNA was extracted with TRIzol reagent (Invitrogen, USA) and reverse transcribed with random primers using the Hiscipt III 1st strand cDNA synthesis kit (Vazyme, Nanjing, China) according to the manufacturer’s instructions. Then, we used SYBR Green Real-Time qPCR analysis (Vazyme, Nanjing, China) to analyze the transcriptional cDNA. The relative expression level of transcripts was normalized to that of the internal control GAPDH and analyzed using the 2^-ΔΔCt method. The forward and reverse primers for GAPDH were GGAGCGAGATCCCTCCAAAAT and GGCTGTTGTCATACTTCTCATGG, respectively. The forward and reverse primers for TEAD1 were ACGTCAAGCCTTTTGTGCAG and CTGAAAATTCCACCAGGCGAAG, respectively.

### 2.9 Western blotting

Cells were harvested after treatment with siRNAs or miRNA and collected by centrifugation after washing with phosphate-buffered saline (PBS) three times. Total protein extracts were prepared in RIPA buffer supplemented with proteinase inhibitors (Solarbio Life Sciences, China). TEAD1 antibody (Abcam, USA), GAPDH, CCND1, CDK4, CDKN1A, CDH1, CDH2, and Vimentin antibody (Proteintech, China) were used for western blot analysis according to the manufacturer’s instructions. Goat Anti-Mouse IgG-HRP (Proteintech, China) and Goat Anti-Rabbit IgG-HRP (Proteintech, China) were used as the secondary antibody. GAPDH was used as a protein loading control. The signals were visualized using the enhanced chemiluminescence (ECL) reagent (4A Biotech, China).

### 2.10 Cell viability assay

Cell viability was evaluated using the Cell Counting Kit-8 (AbMol, USA). HepG2 and Huh-7 cells transfected with siRNAs-TEAD1 were harvested upon reaching 60% confluency. They were then seeded onto 96-well culture plates, with five multiple wells allocated to each group, and 5,000 cells per well. The CCK-8 kit was used to examine the cells at 0 h, 24 h, 48 h, and 72 h after they were incubated at 37°C and 5% CO2.

### 2.11 Flow cytometric analysis of cell cycle

The cell cycle of HepG2 and Huh-7 cells was detected by the Cell Cycle Detection Kit (KeyGen Biotech, China). In brief, cells were collected and fixed in 70% cold ethanol overnight at 4°C. After washing with PBS twice, cells were incubated with PI/RNase A staining buffer for 30 min and subsequently analyzed by Beckman flow cytometry and CytExpert Software.

### 2.12 Transwell assay to detect cell migration and invasion

The migration and invasion of cells were assessed using a Transwell assay. A total of 2 × 10^4 transfected HepG2 and Huh-7 cells were seeded in the upper chamber with or without matrigel and incubated in a serum-free medium, while the lower chamber was incubated in 10% serum medium. After 48 h, the transwell chamber was taken out, fixed with 4% paraformaldehyde for 15 min, and stained with crystal violet for 5 min. Finally, the images were observed and obtained under an optical microscope.

### 2.13 Statistical analysis

Pearson or Spearman correlation coefficients were calculated to evaluate relationships between variables. Real-time fluorescence quantitative PCR and Western blotting were repeated three times. Data analysis was completed using GraphPad Prism 9 software. The student’s t-test was used for the comparison between the two groups, and Two-way ANOVA was used for the comparison between multiple groups to determine the significance; statistical significance was determined at p < 0.05, with *p < 0.05, **p < 0.01, ***p < 0.001, and ns indicating not significant. The data are expressed as Mean ± SD.

## 3 Results

### 3.1 Pan-cancer expression pattern and clinical prognostic significance of TEAD1

**Figure 1:**
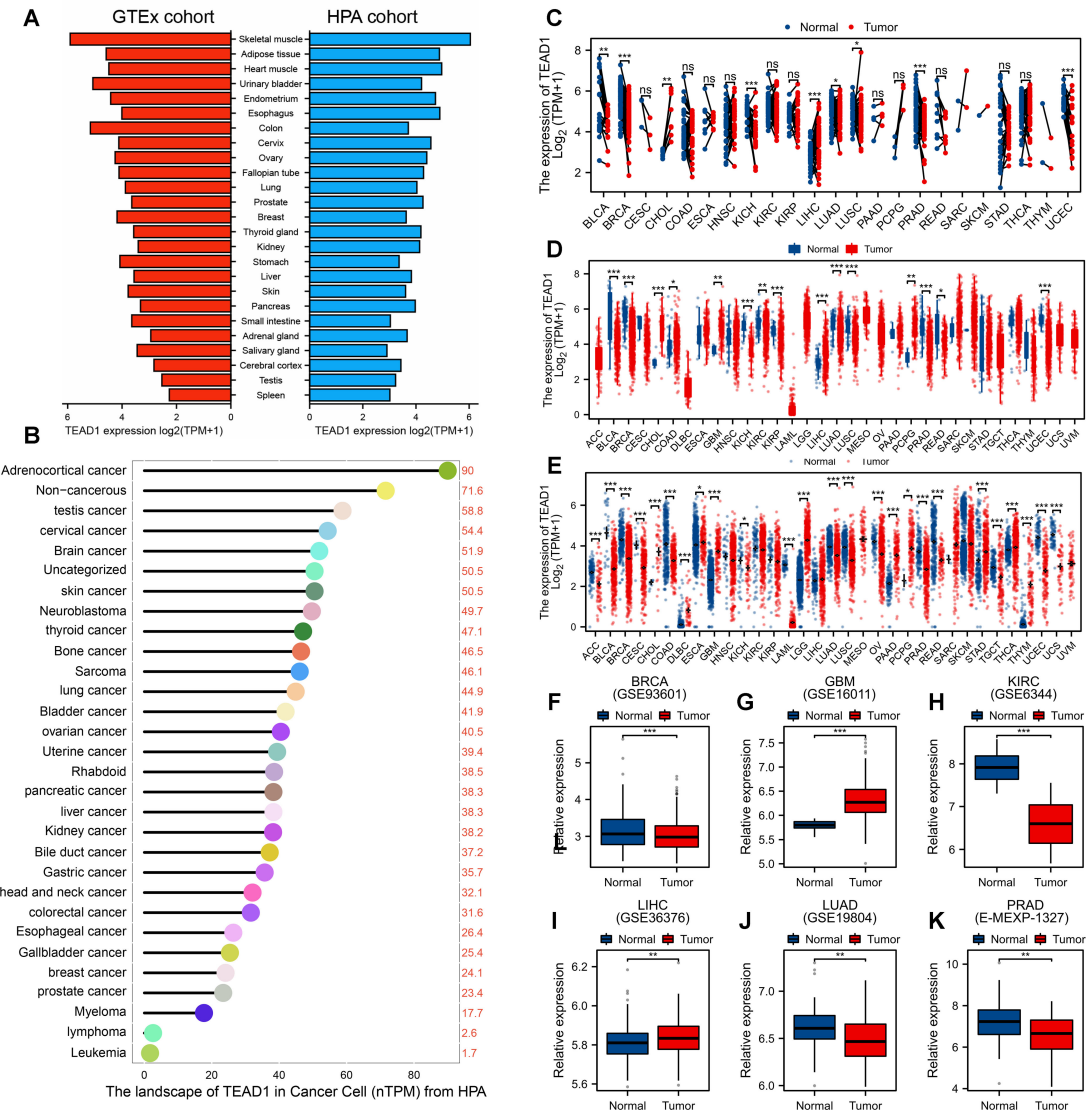
Expression of TEAD1 in human normal tissues and cancers. **(A)** Expression level of TEAD1 in human normal tissues (HPA+GTEx datasets). **(B)** TEAD1 expression in human cancer cell lines. **(C)** Evaluation of differential expression of TEAD1 based on TCGA paired samples. **(D)** Differential expression analysis based on all cancer and normal samples from TCGA. **(E)** Evaluation of TEAD1 mRNA expression levels by combining TCGA and GTEx datasets. **(F-K)** The differential expression of TEAD1 was verified based on multiple cancer datasets in GEO. *p < 0.05; **p < 0.01; ***p < 0.001; ns, no significance.

TEAD1 expression in normal tissues is ubiquitous, expressed to varying degrees in almost all tissues, rather than being organ-specific. As shown in **Figure 1A**, its presence is relatively high in skeletal muscle and adipose tissue. Expression profiling analysis of cancer cell lines showed that TEAD1 was highly expressed in adrenocortical carcinoma, non-cancerous cancers, and testicular cancer cell lines (**Figure 1B**). Differential expression analysis based on TCGA paired samples showed that TEAD1 was mainly highly expressed in cholangiocarcinoma (CHOL), LIHC, and lung squamous cell carcinoma (LUSC), while significantly lowly expressed in cancers such as bladder urothelial carcinoma (BLCA), breast invasive carcinoma (BRCA), and kidney chromophobe (KICH) (**Figure 1C**). Differential expression analysis based on all cancer and normal samples from TCGA also confirmed the high expression of TEAD1 in cancers including CHOL, glioma (GBM), and LIHC (**Figure 1D**). To expand the sample size and obtain more reliable results, we integrated normal samples from the GTEx database and observed widespread dysregulation of TEAD1 in more than four-fifths of cancer types (**Figure 1E**). These results were validated by multiple GEO datasets (**Figures 1F–K**). In addition, we evaluated the correlation between TEAD1 and the clinical stage of cancer using the TISIDB database. We found that TEAD1 expression was significantly associated with higher clinical stages of multiple cancers, including adrenocortical carcinoma (ACC), BLCA, head and neck squamous cell carcinoma (HNSC), and kidney renal clear cell carcinoma (KIRC) (**Supplementary Figures 1A–L**). Prognostic analysis showed a significant correlation between TEAD1 and the prognosis of ACC, BLCA, KICH, and KIRC. In particular, high TEAD1 expression in ACC and BLCA patients was significantly associated with shorter OS, DSS, and PFI. In addition, it was also associated with shorter OS in BRCA patients, shorter DSS in KICH patients, and shorter DSS and PFI in LUSC patients. In addition, low TEAD1 expression in KIRC patients was significantly associated with shorter OS, DSS, and PFI (**Supplementary Figure 1M**).

**Figure 2:**
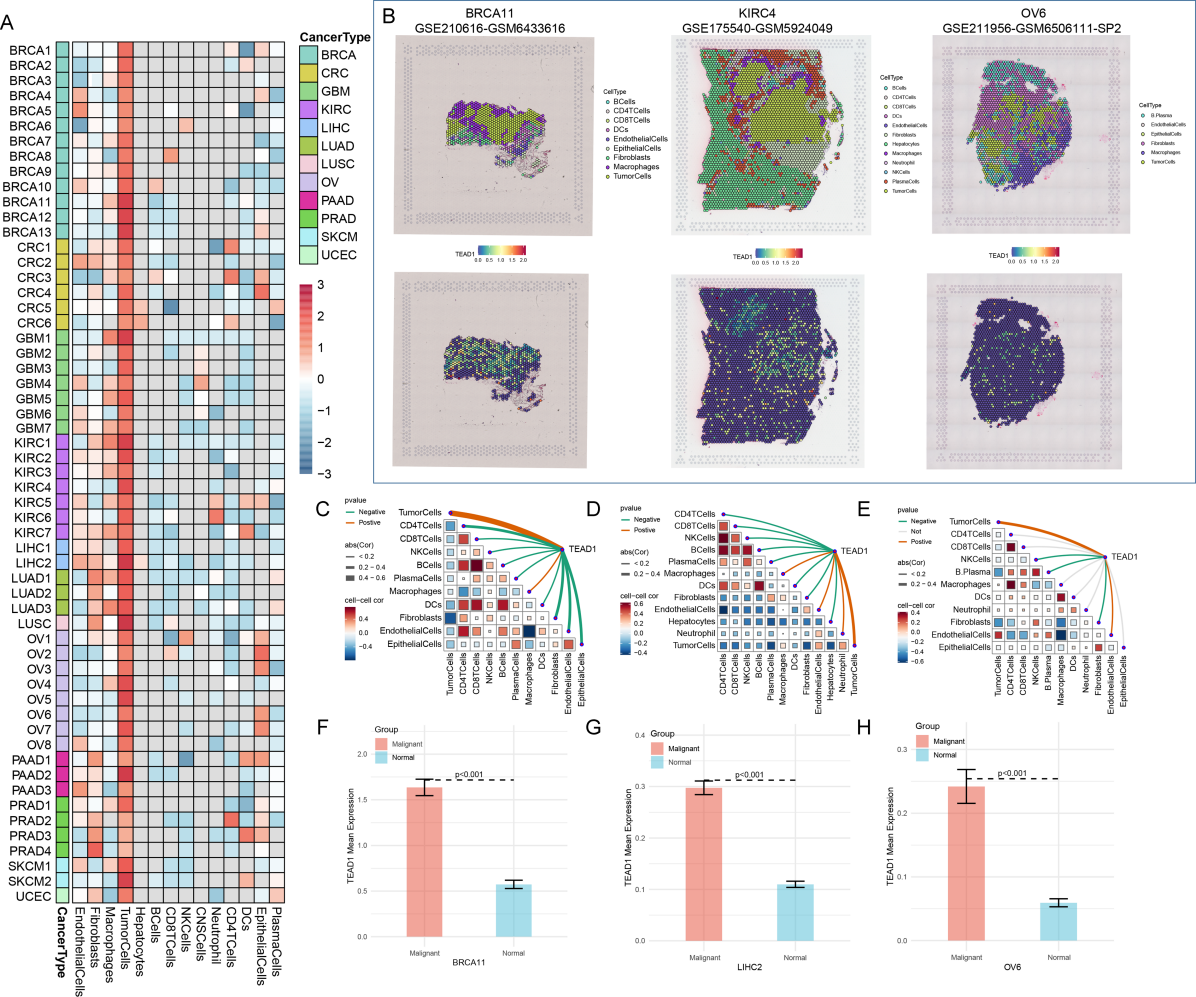
TEAD1 was significantly associated with tumor cells. **(A)** Single cell expression of TEAD1 in pan-cancer (TISCH2 database). **(B)** UMAP localization map of TEAD1 in BRCA, LIHC, and OV. **(C–E)** Evaluation of the correlation of each cell type in the TEAD1 gene expression. **(F–H)** Evaluation of TEAD1 gene expression differences between malignant and normal cells.

In addition, we evaluated the expression of TEAD1 in pan-cancer at spatial transcriptome resolution. We observed that TEAD1 expression in tumor cells was dominant in multiple cancer types, including BRCA, CRC, and LIHC (**Figure 2A**). Further localization analysis also showed that TEAD1 was significantly highly expressed in tumor cells in BRCA, KIRC, and ovarian serous cystadenocarcinoma (OV) (**Figure 2B**). Highly consistent with the localization results, the expression level of TEAD1 was significantly positively correlated with the content of tumor cells in the spot (**Figures 2C–E**). In addition, TEAD1 was more highly expressed in malignant areas compared to non-malignant areas (**Figures 2F–H**). These results highlight the important role of TEAD1 in various tumors.

### 3.2 The epigenetic variations of TEAD1 in pan-cancer

**Figure 3:**
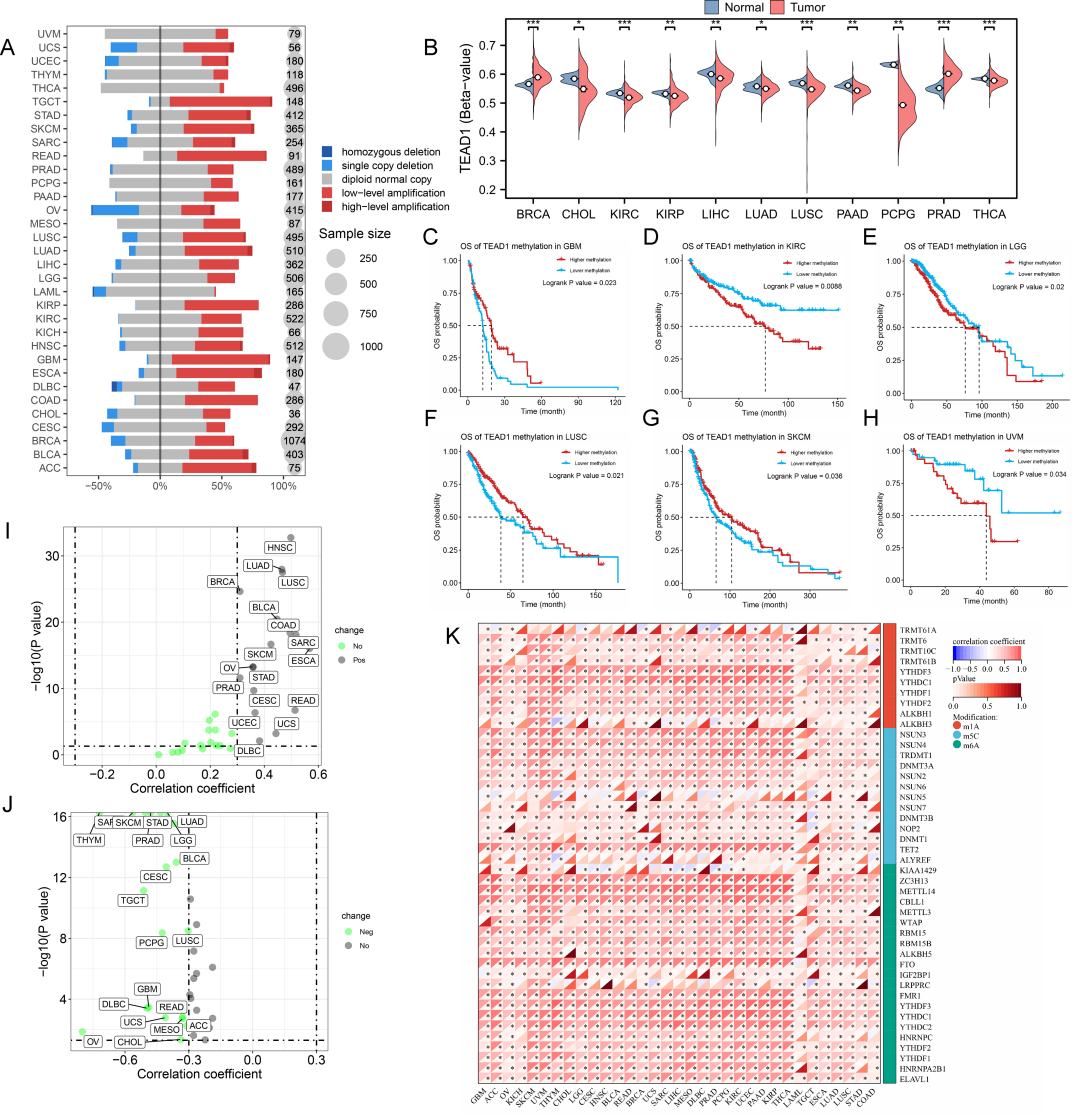
The epigenetic variations of TEAD1 in pan-cancer. **(A)** Copy number variation levels of TEAD1 in pan-cancer. **(B)** Differential methylation levels of TEAD1 in normal and tumor tissues in multiple cancer types. **(C-H)** The relationship between methylation of TEAD1 and prognosis in GBM, KIRC, LGG, LUSC, SKCM and UVM. **(I)** The relationship between copy number variation of TEAD1 and mRNA expression of TEAD1 in pan-cancer. **(J)** The relationship between methylation of TEAD1 and mRNA expression of TEAD1 in pan-cancer. **(K)** Correlation between TEAD1 and RNA-modifying genes in pan-cancer. *p < 0.05; **p < 0.01; ***p < 0.001; ns, no significance.

To reveal the mechanisms leading to dysregulated TEAD1 expression, we evaluated the CNV and methylation levels of TEAD1 in pan-cancer. We observed more copy number amplifications in multiple tumor types, including ACC, BLCA, GBM, and rectum adenocarcinoma (READ), while more copy number losses were observed in OV (**Figure 3A**). Methylation analysis showed that compared with normal tissues, lower methylation levels were observed in multiple tumor samples, including CHOL, KIRC, kidney renal papillary cell carcinoma (KIRP), LIHC, and lung adenocarcinoma (LUAD), while higher methylation levels were observed in BRCA and PRAD (**Figure 3B**). Survival analysis showed that patients with high methylation levels of TEAD1 had better prognoses in GBM, LUSC, and skin cutaneous melanoma (SKCM), while the opposite was true in KIRC, lower grade glioma (LGG), and uveal melanoma (UVM) (**Figures 3C–H**). We also analyzed the correlation between TEAD1 CNV, methylation levels, and mRNA expression. The results showed that in various cancers, TEAD1 mRNA expression was significantly positively correlated with its CNV (**Figure 3I**) and negatively correlated with its methylation level (**Figure 3J**). In addition, TEAD1 was also significantly associated with genes associated with RNA methylation modification in pan-cancer (**Figure 3K**). These results emphasize that epigenetic variations in TEAD1 may mediate its mRNA expression and participate in cancer progression.

### 3.3 Single-cell analysis reveals a link between TEAD1 and LIHC malignant cells

**Figure 4:**
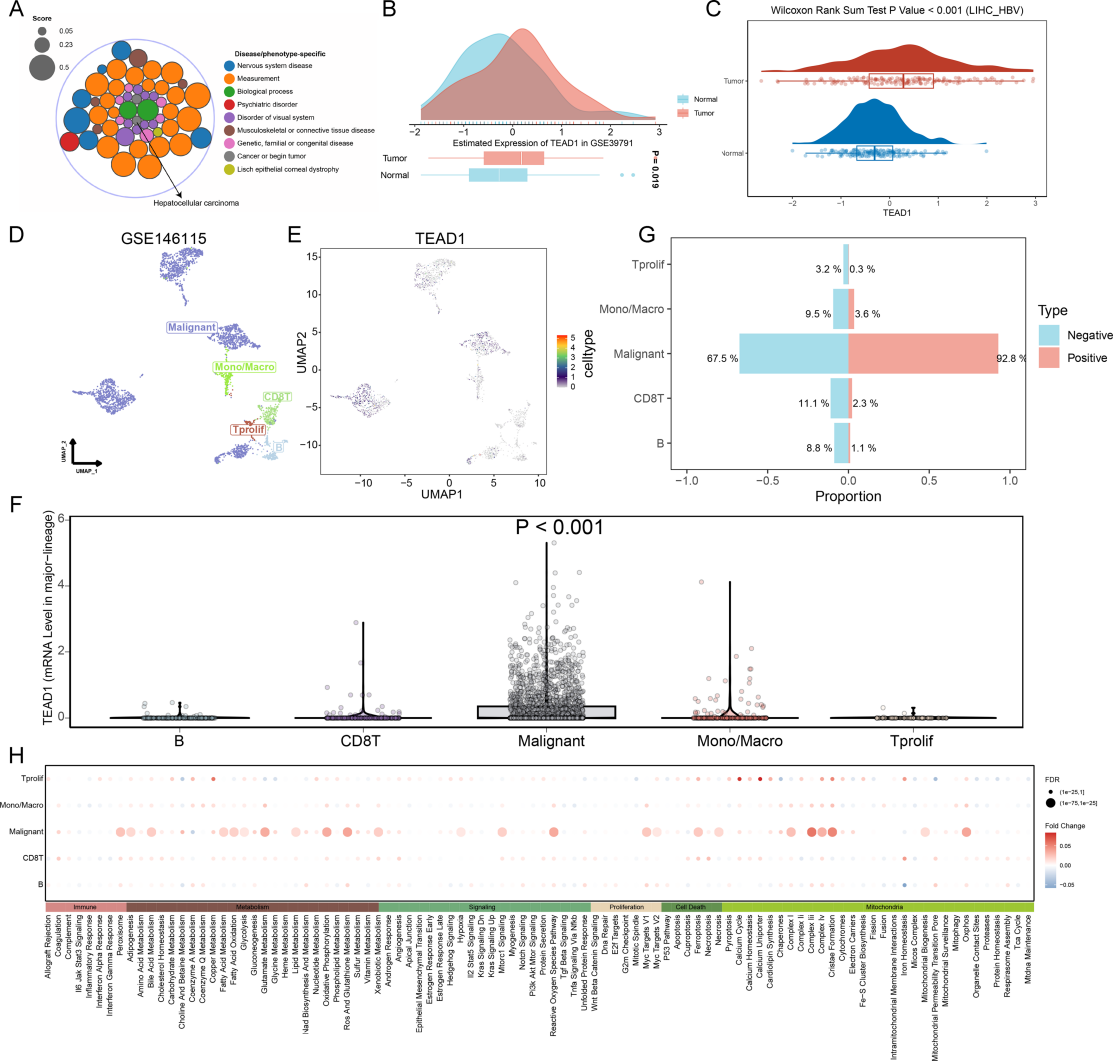
Single-cell analysis reveals a link between TEAD1 and LIHC malignant cells. **(A)** Based on the Open the Targets of Platform (https://platform.opentargets.org/) link between TEAD1 and disease were analyzed. **(B)** Evaluation of TEAD1 expression in hepatocellular carcinoma based on GEO datasets. **(C)** TEAD1 protein expression in LIHC based on the Clinical Proteomic Tumor Analysis Consortium database. **(D)** UMAP of major cell lineages in the single-cell dataset LIHC_GSE146115. **(E)** UMAP localization map of TEAD1 in the single-cell dataset LIHC_GSE146115. **(F)** Evaluation of TEAD1 gene expression differences between different cells based on single cell dataset LIHC_GSE146115. **(G)** Evaluation of the proportion of each cell type in the TEAD1 gene expression positive group and negative group based on the single cell dataset LIHC_GSE146115. **(H)** Evaluation of pathway differences in each cell type between the TEAD1 gene expression positive group and the negative group based on the single cell dataset LIHC_GSE146115.

Based on the Open Targets platform (https://platform.opentargets.org/), we analyzed the connection between TEAD1 and disease. We observed a significant correlation between TEAD1 and LIHC in cancer types (**Figure 4A**). Therefore, we focused on the association between TEAD1 and LIHC. We first verified the significantly high expression of TEAD1 in hepatocellular carcinoma in additional GEO datasets (**Figure 4B**). In addition, at the protein level, we also observed significantly high expression of TEAD1 in LIHC tumor samples (**Figure 4C**). Single-cell analysis results showed that TEAD1 was significantly highly expressed in malignant cells of LIHC (**Figures 4D–F**). In addition, we also observed that in the LIHC_GSE146115 dataset, the proportion of malignant cells in the TEAD1-positive expression group was much higher than that in the TEAD1-negative expression group (**Figure 4G**). Pathway analysis showed that in malignant cells, Metabolism and Mitochondria-related biological pathways scored higher in the TEAD1-positive group (**Figure 4H**).

### 3.4 Immunological characteristics of TEAD1 in LIHC

**Figure 5:**
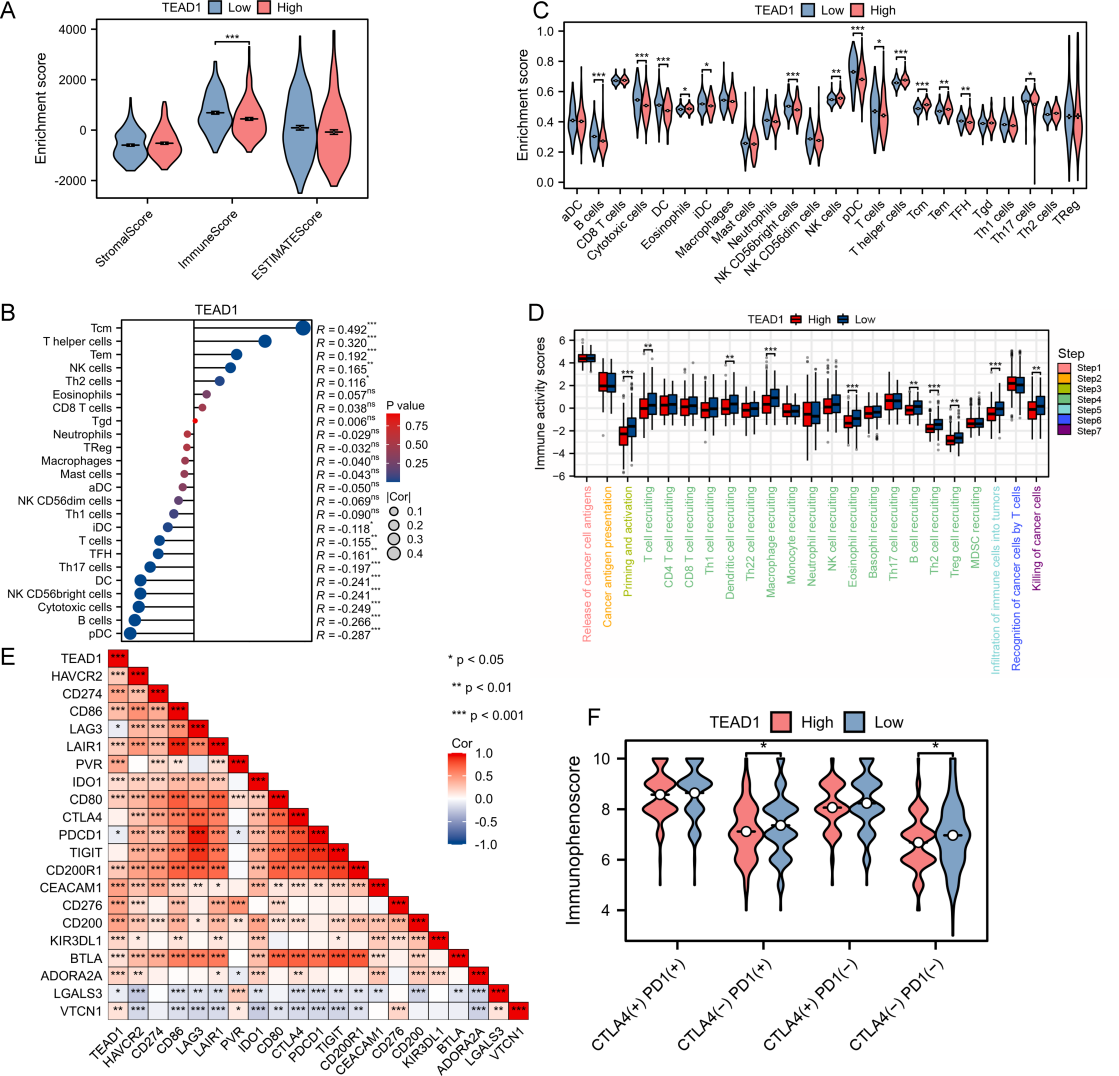
Immunological characteristic of TEAD1 in LIHC. **(A)** Differences in the immune scores between high- and low-TEAD1 groups. **(B)** Correlation between TEAD1 and 24 immune cell in LIHC. **(C)** Differences in Enrichment scores among the 24 immune cells between high- and low-TEAD1 groups. **(D)** Differences in the various steps of the cancer immunity cycle between high- and low-TEAD1 groups. **(E)** Correlation between immune checkpoints and TEAD1 in LIHC. **(F)** Differences in immune checkpoint therapy between high- and low-TEAD1 groups. *p < 0.05; **p < 0.01; ***p < 0.001; ns, no significance.

TEAD1 was significantly negatively correlated with the immune score in LIHC (**Figure 5A**). Immune cell infiltration analysis based on the CIBERSORT algorithm showed that TEAD1 was significantly positively correlated with Tcm cells (R = 0.492, P < 0.001) and T helper cells (R = 0.320, P < 0.001), but significantly negatively correlated with pDC cells (R = -0.287, P < 0.001) and B cells (-0.266, P < 0.001) (**Figure 5B**). In addition, immune cell infiltration analysis based on the ssGSEA algorithm also showed that the TEAD1 low expression group had higher B cell enrichment scores, DC cell enrichment scores, and T cell enrichment scores, while higher T helper cell enrichment scores and Tcm enrichment scores were observed in the TEAD1 high expression group (**Figure 5C**). In addition, we analyzed the anti-cancer immune status of the TEAD1 high and low expression groups at seven different stages of the tumor immune cycle (**Figure 5D**). We observed that the activity of most steps in the TEAD1 high expression group was downregulated, including priming and activation (step 3), immune cell infiltration into tumors (step 5), and immune cell trafficking to tumors (step 4) (T cell recruitment, dendritic cell recruitment, macrophage recruitment, eosinophil recruitment, B cell recruitment, Th2 cell recruitment, Treg cell recruitment). The downregulation of the activity of these steps may reduce the infiltration level of effector immune cells. It is worth noting that the TEAD1 low expression group has higher infiltration of immune cells into tumors and killing of cancer cells activity. Correlation analysis showed that TEAD1 was significantly positively correlated with multiple immune checkpoints in LIHC, including CD274, D86, and CD276 (**Figure 5E**). Immunotherapy analysis showed that patients with lower TEAD1 expression in LIHC benefited more from PD1 therapy (**Figure 5F**).

### 3.5 Functional enrichment analysis of TEAD1 and construction of a prognostic model based on disulfidptosis in LIHC

**Figure 6:**
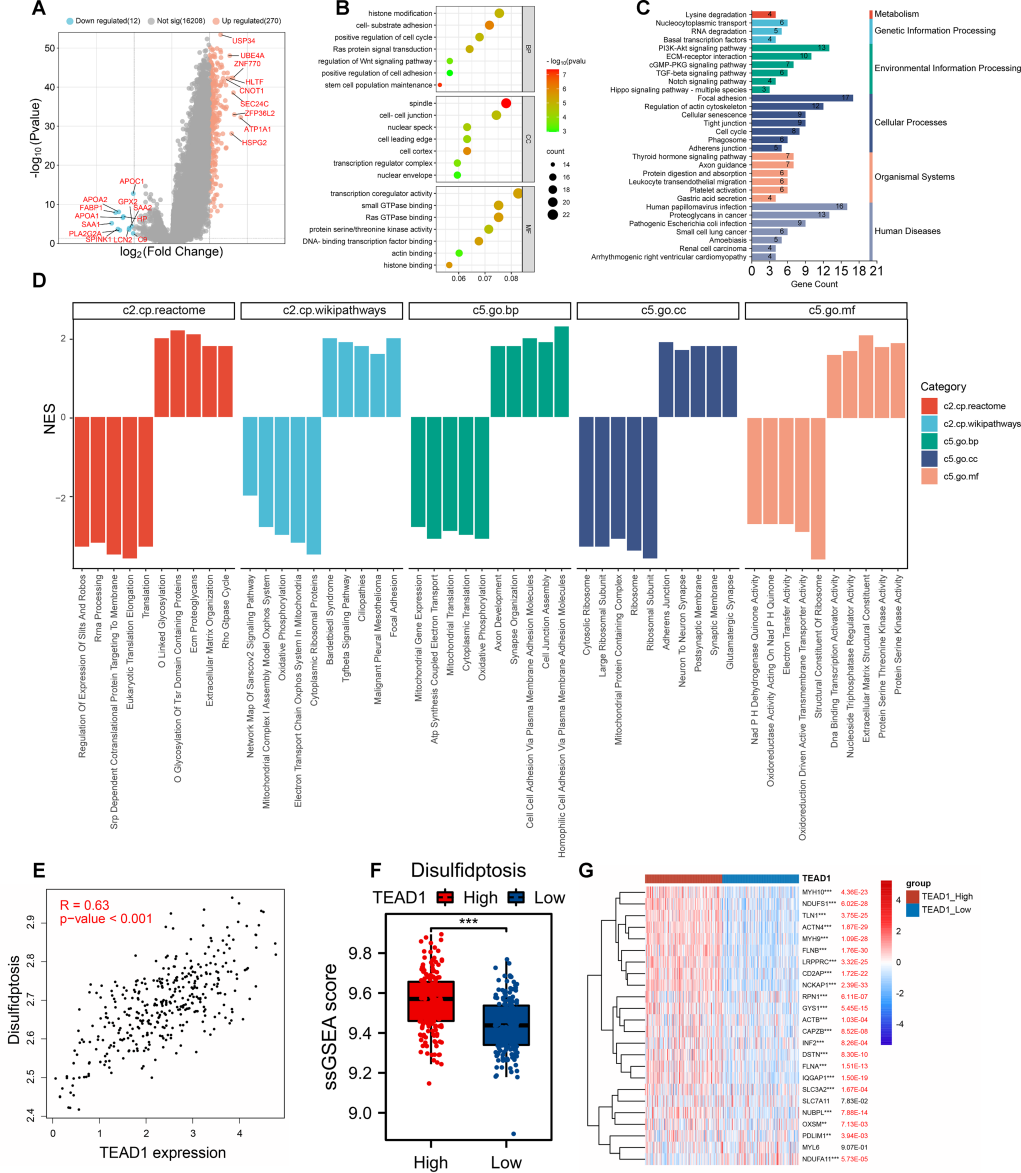
Functional enrichment analysis of TEAD1 in hepatocellular carcinoma. **(A)** Volcano map of differential genes in TEAD1 high and low expression groups. **(B, C)** GO and KEGG functional enrichment analysis. **(D)** GSEA enrichment analysis. **(E)** Correlation between TEAD1 and disulfidptosis in LIHC. **(F)** The disulfidptosis score of LIHC patients were evaluated based on ssGESA in high- and low-TEAD1 groups. **(G)** Correlation between TEAD1 and disulfidptosis -related genes in LIHC. **p < 0.01; ***p < 0.001.

To explore the potential molecular mechanism of TEAD1 in LIHC, we first grouped LIHC samples according to the median expression value of TEAD1 and performed differential analysis. A total of 270 upregulated genes and 12 downregulated genes were identified (**Figure 6A**). We selected 270 upregulated genes for GO and KEGG enrichment analysis. The results showed that GO-BP functional enrichment analysis showed that differentially expressed genes were mainly significantly enriched in pathways such as histone modification, cell-matrix adhesion, positive regulation of the cell cycle, and regulation of the Wnt signaling pathway. For GO-CC, differentially expressed genes were mainly enriched in spindles, cell-cell junctions, and cell leading edges. For GO-MF, differentially expressed genes were mainly enriched in transcriptional co-regulatory activity, small GTPase binding, and Ras GTPase binding (**Figure 6B**). KEGG enrichment analysis (**Figure 6C**) showed that differentially expressed genes were mainly enriched in multiple cancer-related sets in human diseases. In addition, significant enrichment of multiple cancer-related pathways such as the PI3K-Akt signaling pathway, ECM-receptor interaction, and TGF-β signaling pathway was observed. Gene set enrichment analysis (GSEA) based on multiple datasets showed that TEAD1 was mainly associated with cell adhesion, organization of the extracellular matrix, signal transduction, neural development and function, and assembly and maintenance of cell junctions and synapses (**Figure 6D**).

**Figure 7:**
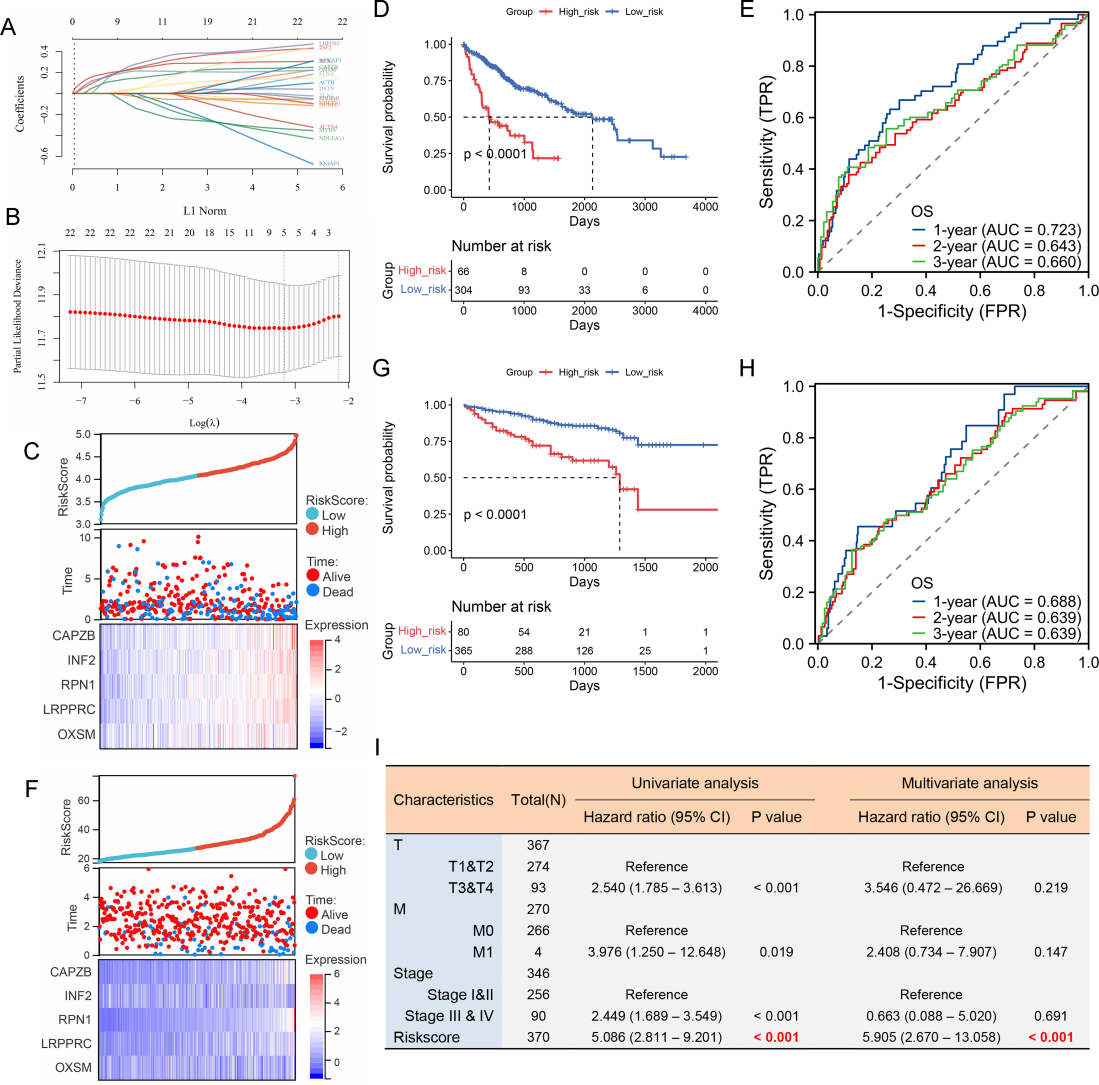
A LIHC prognostic model was constructed using 22 disulfidptosis-related genes that were significantly correlated with TEAD1. **(A)** LASSO coefficient profiles for 22 disulfidptosis -related genes in the TCGA cohort. **(B)** Partial likelihood deviations were plotted versus log(λ) using a LASSO Cox regression model. **(C–E)** Risk factor heat map, survival analysis and ROC analysis of prognostic model in TCGA dataset. **(F–H)** Risk factor heat map, survival analysis and ROC analysis of prognostic model in ICGC dataset. **(I)** The univariate and multivariate Cox regression analysis showed that this prediction model was an independent prognostic factor for LIHC.

Disulfidptosis is a newly discovered cell death mechanism caused by cytoskeletal collapse caused by disulfide stress. Using the correlation analysis module in GEPIA2 (http://gepia2.cancer-pku.cn/), we observed a significant positive correlation between TEAD1 and disulfidptosis in LIHC (R=0.63, P<0.001) (**Figure 6E**). In addition, using the ssGSEA algorithm, we calculated the disulfidptosis score of TCGA-LIHC patients, and we observed higher disulfidptosis scores in the TEAD1 high expression group (**Figure 6F**). In addition, there was a significant positive correlation between TEAD1 and 24 disulfidptosis-related genes in LIHC (**Figure 6G**). We further constructed a prognostic model for hepatocellular carcinoma using 22 disulfide apoptosis genes that were significantly positively correlated with TEAD1 (**Figures 7A, B**). The lambda.min of LASSOS cox was 0.0404, and the model formula was Riskscore=(0.1088)*CAPZB+(0.1654)*INF2+(0.1927)*RPN1+(0.1584)*LRPPRC+(0.1401)*OXSM. Survival analysis showed that patients in the high riskScore group had a shorter survival time, and the AUCs of the model predicting 1-, 2-, and 3-year survival rates were 0.723, 0.643, and 0.660, respectively (**Figures 7C–E**), indicating that the model has good predictive performance. In addition, we used the liver cancer dataset of ICGC to validate our model, and the results showed that the high riskScore group had a poor prognosis. The AUCs of this model for predicting the 1-year, 2-year, and 3-year survival rates of ICGC liver cancer patients were 0.688, 0.639, and 0.639, respectively (**Figures 7F–H**), which showed good predictive performance. In addition, the results of univariate and multivariate Cox regression analysis showed that this prediction model was an independent prognostic factor for LIHC (**Figure 7I**).

### 3.6 TEAD1 regulates LIHC cell proliferation and cell cycle

**Figure 8:**
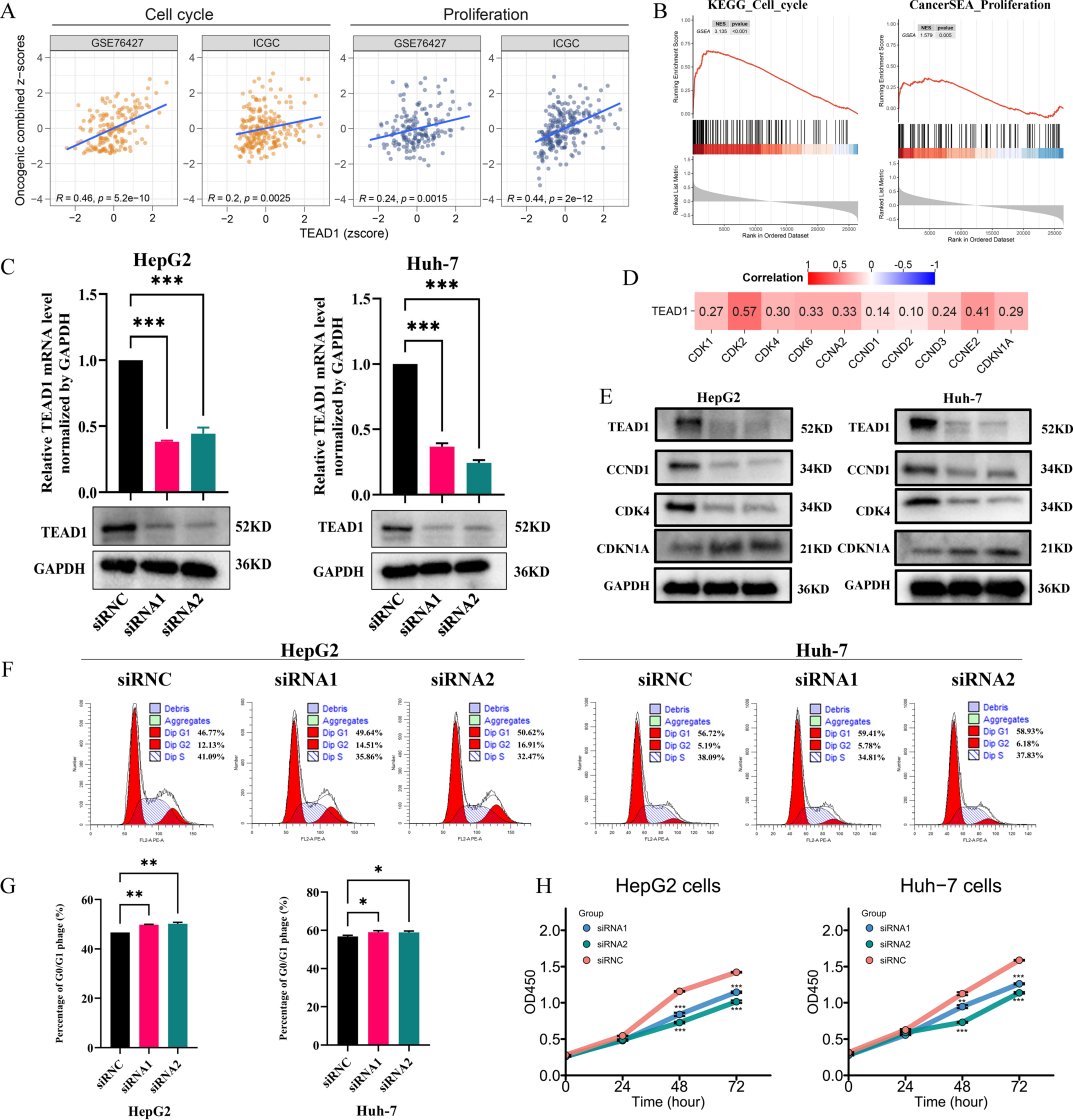
TEAD1 regulates LIHC cell proliferation and cell cycle. **(A, B)** Functional enrichment analysis based on GSVA and GSEA. **(C)** After HepG2 and Huh-7 cells were transfected with siRNA1/2 or scrambled control (siRNC), mRNA and protein of TEAD1 was detected. **(D)** Heatmap of the correlation between TEAD1 expression and cell cycle related genes. **(E)** The protein level of cell cycle related genes with/without TEAD1 knockdown in HepG2 and Huh-7 cells. **(F, G)** Flow cytometry detected the cell cycle distribution in HepG2 and Huh-7 cells. **(H)** CCK-8 assay with/without TEAD1 depletion in HepG2 and Huh-7 cells. The data are presented as the mean ± SD. from three independent experiments. ***P < 0.001, **P < 0.01, *P < 0.05.

Functional enrichment analysis based on GSVA and GSEA showed that TEAD1 was significantly positively correlated with cell cycle and cell proliferation in LIHC (**Figures 8A, B**). To further verify this result, we selected HepG2 and Huh-7 cell lines for cell function experiments. As shown in **Figure 8C**, the mRNA expression and protein expression of TEAD1 were significantly knocked down in both cells after siRNA transfection. Correlation analysis based on TCGA-LIHC showed that TEAD1 was positively correlated with cell cycle-related genes, including CDK2 (r = 0.57), CDK4 (r = 0.30), CDK6 (r = 0.33), and CCNE2 (r = 0.41) (**Figure 8D**). Western blot analysis showed that after knocking down TEAD1, the expression of CCND1 and CDK4 was significantly reduced, while the expression of CDKN1A was significantly increased (**Figure 8E**). Furthermore, we examined the effect of TEAD1 knockdown on the cell cycle and observed G0/G1 phase arrest in both cell lines (**Figures 8F, G**). In addition, CCK-8 assay showed that after TEAD1 knockdown, cell proliferation ability was significantly reduced (**Figure 8H**).

### 3.7 TEAD1 affects the migration and invasion in LIHC

**Figure 9:**
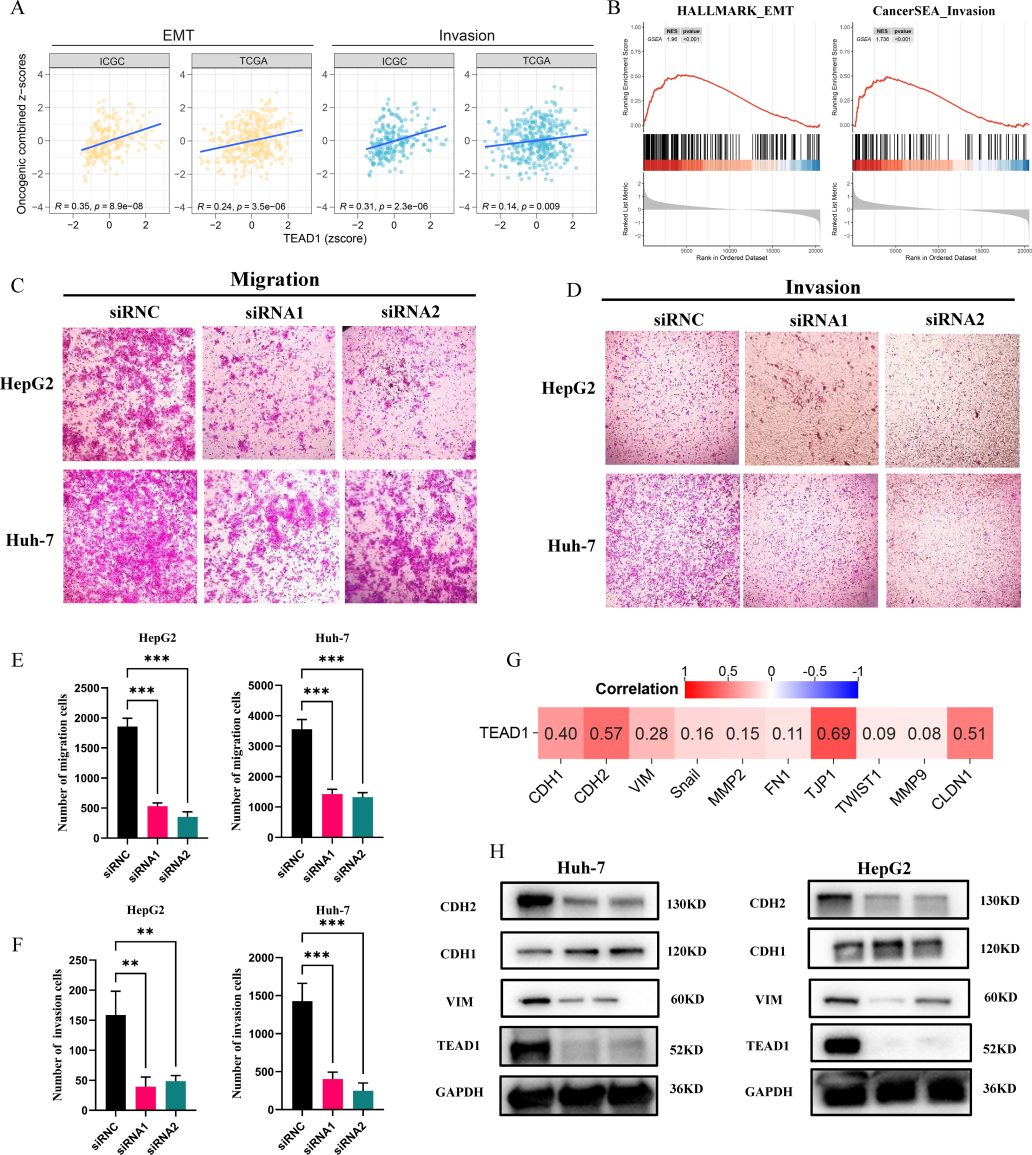
TEAD1 affects the migration and invasion in LIHC. **(A, B)** Functional enrichment analysis based on GSVA and GSEA. **(C–F)** Transwell assay was conducted used for HepG2 and Huh-7cell migration and invasion. **(G)** Heatmap of the correlation between TEAD1 expression and EMT and invasion related genes. **(H)** The protein level of EMT related genes with/without TEAD1 knockdown in HepG2 and Huh-7 cells. The data are presented as the mean ± SD. from three independent experiments. ***P < 0.001, **P < 0.01, *P < 0.05.

GSVA and GSEA analysis showed that TEAD1 was significantly correlated with the epithelial-mesenchymal transition (EMT) and invasion pathways of LIHC (**Figures 9A, B**). It is well known that the programmed activation of EMT is involved in the metastasis of epithelial malignant tumor cells (21). The relationship between TEAD1 expression and tumor metastasis was further verified. The results of Transwell migration assay and matrigel invasion assay (**Figures 9C–F**) confirmed that reducing TEAD1 expression could inhibit the migration and invasion of LIHC cells. Correlation analysis based on TCGA-LIHC showed that TEAD1 was positively correlated with EMT proteins, including CDH2 (r=0.57), VIM (r=0.28), CLDN1 (r=0.51), and TJP1 (r=0.69) (**Figure 9G**). Interestingly, western blot results showed that low expression of TEAD1 was accompanied by an increase in CDH1 and a decrease in the expression levels of VIM and CDH2 (**Figure 9H**).

## 4 Discussion

LIHC is a highly aggressive malignancy characterized by metabolic heterogeneity (22). Despite the implementation of multidisciplinary diagnostic and therapeutic strategies, including surgical resection, radical hepatectomy, targeted therapies, and immunotherapies, the overall survival (OS) rates for patients with advanced LIHC remain disappointingly low (23, 24). Consequently, there is an urgent need for innovative biomarkers that can predict prognosis, facilitate risk stratification, and identify therapeutic targets for individuals diagnosed with LIHC. TEAD1/Tef-1, encoded by TEAD1 gene, has garnered extensive attention due to its critical role in multiple cancers (25–27). Previous studies have demonstrated that TEAD1 can function as either a promoter or a suppressor of tumorigenesis, depending on the specific cancer context (28–30). Therefore, a deeper comprehension of the mechanisms through which TEAD1 participates in oncogenesis is highly desirable. This study thoroughly investigates the multifaceted roles of the TEAD1 gene in cancer biology, particularly in LIHC. Our results indicate that TEAD1expression levels vary significantly across different cancer types. Furthermore, we found that TEAD1 expression was closely associated with clinical outcomes across multiple cancers; these findings are consistent with existing literature. In addition, our study also found that the epigenetic changes of TEAD1 are highly heterogeneous in multiple cancers, and its abnormal methylation and CNV are associated with poor prognosis in multiple cancers. This finding emphasizes the importance of epigenetics in TEAD1 regulation and may provide new targets for personalized treatment of cancer.

In LIHC, the significant correlation between TEAD1and malignant cells highlights the multifaceted roles that TEAD1 may play in hepatocellular carcinoma, including its potential as both a biomarker and therapeutic target. The tumor immune microenvironment is intricately linked to the initiation and progression of tumors (31, 32). We found that the expression of TEAD1 in LIHC was significantly negatively correlated with the immune score, suggesting its critical role in suppressing tumor immune responses. In addition, TEAD1 was associated with the infiltration level of specific subsets of immune cell, thereby influencing the composition of the tumor microenvironment. We observed that the high TEAD1 expression group exhibited active downregulation at multiple stages of the tumor immune cycle, which may lead to reduce the infiltration levels of effector immune cells. Notably, there was a positively correlation between TEAD1 and multiple immune checkpoints, patients with low expressions of TEAD1 appeared to benefit more from PD1 treatment. This indicates that the level of TEAD1 expression could serve as a predictive biomarker for immunotherapy response. These findings elucidate the potential role of TEAD1 in modulating both the LIHC immune microenvironment and responses to immunotherapy. They also provide new avenues for future research aimed at gaining deeper insights into the mechanisms by which TEAD1 operates in LIHC and developing novel therapeutic strategies.

The TEAD family of transcription factors, which are evolutionarily conserved across species, exhibit minimal intrinsic transcriptional activity and require the presence of coactivators to effectively induce target genes (33–35). YAP/TAZ, as core downstream components of the Hippo pathway, have emerged as the most well-established activators of TEAD (36, 37). The YAP/TAZ-TEAD complex has been identified as a significant driver in cancer progression, influencing tumorigenesis, growth, EM, metastasis, and drug resistance (38–42). In this study, we found that TEAD1 was significantly correlated with the expression of genes involved in the cell regulation, cell proliferation, EMT processes, and invasion pathways through functional enrichment analysis. Furthermore, our experimental results demonstrated that knockdown of TEAD1 led to a reduction in the proliferation, migration, and invasion capabilities of LIHC cells. Interestingly, In contrast to correlation analysis based on TCGA-LIHC, western blot results showed that low expression of TEAD1 was accompanied by an increase in CDH1. This discrepancy may reflect post-translational modifications influenced by TEAD1, warranting further investigation into its role in protein regulation.

We further developed a prognostic model incorporating disulfidptosis-related genes, which demonstrated robust predictive performance in LIHC patients. Unlike network-based approaches such as mRank that identify biomarker modules within gene regulatory networks (43), our lasso-Cox-based model (44, 45) uniquely integrates the novel cell death mechanism of disulfidptosis with TEAD1 activity, providing mechanistic insights into HCC prognosis. This model not only offers a clinically relevant prognostic tool but also suggests new avenues for understanding TEAD1’s functional mechanisms in LIHC.

Several limitations of our study should be noted. First, while our *in vitro* findings are compelling, they require validation in animal models and clinical samples to establish translational relevance. Second, the precise mechanisms underlying TEAD1’s apparent regulation of CDH1 and its potential role in post-translational modifications remain to be elucidated. Third, although we observed associations between TEAD1 and the tumor immune microenvironment, the specific immunomodulatory mechanisms merit further investigation. Finally, while our prognostic model shows promise, its generalizability across diverse patient populations and disease stages requires additional validation through multicenter studies and multi-omics integration (e.g., incorporating methylation and proteomic data).

## 5 Conclusions

In summary, our study not only elucidates the multifaceted roles of TEAD1 in LIHC but also offers new avenues for future research. Subsequent investigations should concentrate on the molecular mechanisms underlying TEAD1`s function, its potential in the tumor immune microenvironment, and its potential as a therapeutic target. Through these endeavors, we aspire to develop more effective treatment strategies for patients with LIHC.

## Data Availability

The datasets presented in this study are openly available in online repositories. Detailed information, including repository names and accession numbers, is provided within the article and/or **Supplementary Material**.

## Supplementary material

Supplementary Figure 1
**(A-L)** Correlation between TEAD1 expression and clinical stages. **(M)** Forest plots show univariable Cox regression analysis for OS, DSS, DFI and PFI of TEAD1 expression in 33 TCGA cancer types.
