# Supplementary figures and images for: Dissecting the multi-omics landscape of TEAD1 in hepatocellular carcinoma: cycle regulation and metastatic potential
Source: Front Immunol. 2025 Jun 5;16:1567969. doi: 10.3389/fimmu.2025.1567969 (PMC12177558; doi:10.3389/fimmu.2025.1567969)

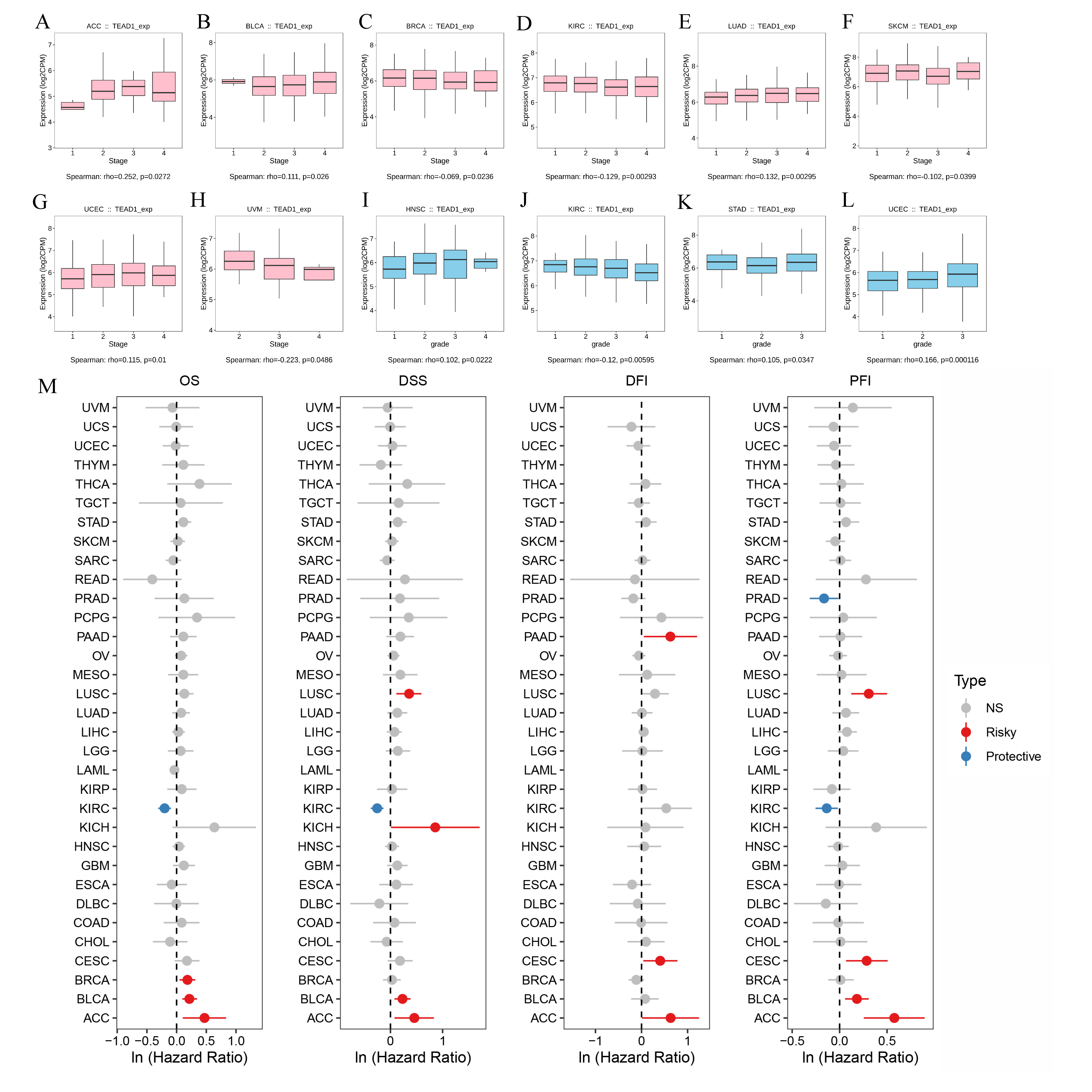

Supplement: Supplementary Figure 1 — (A-L) Correlation between TEAD1 expression and clinical stages. (M) Forest plots show univariable Cox regression analysis for OS, DSS, DFI and PFI of TEAD1 expression in 33 TCGA cancer types. [file Image1.tif]
