# Supplementary material for: Dissecting the multi-omics landscape of TEAD1 in hepatocellular carcinoma: cycle regulation and metastatic potential
Source: Front Immunol. 2025 Jun 5;16:1567969. doi: 10.3389/fimmu.2025.1567969 (PMC12177558; doi:10.3389/fimmu.2025.1567969)
Supplement: Supplementary file 14 [file Presentation1.pptx]

## Slide 1
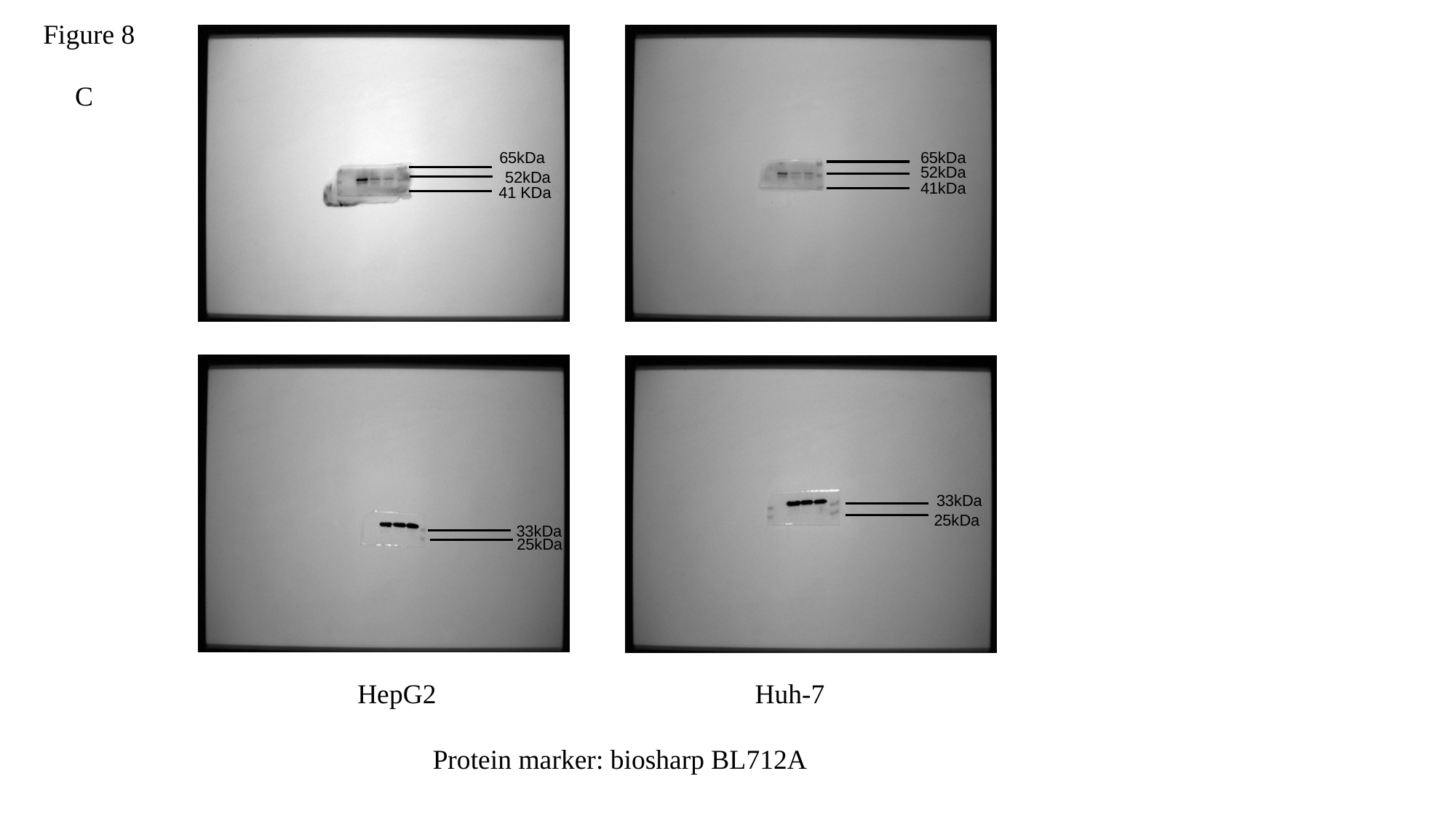

Figure 8
C
65kDa
65kDa
52kDa
52kDa
41kDa
41 KDa
33kDa
25kDa
33kDa
25kDa
HepG2
Huh-7
Protein marker: biosharp BL712A

## Slide 2
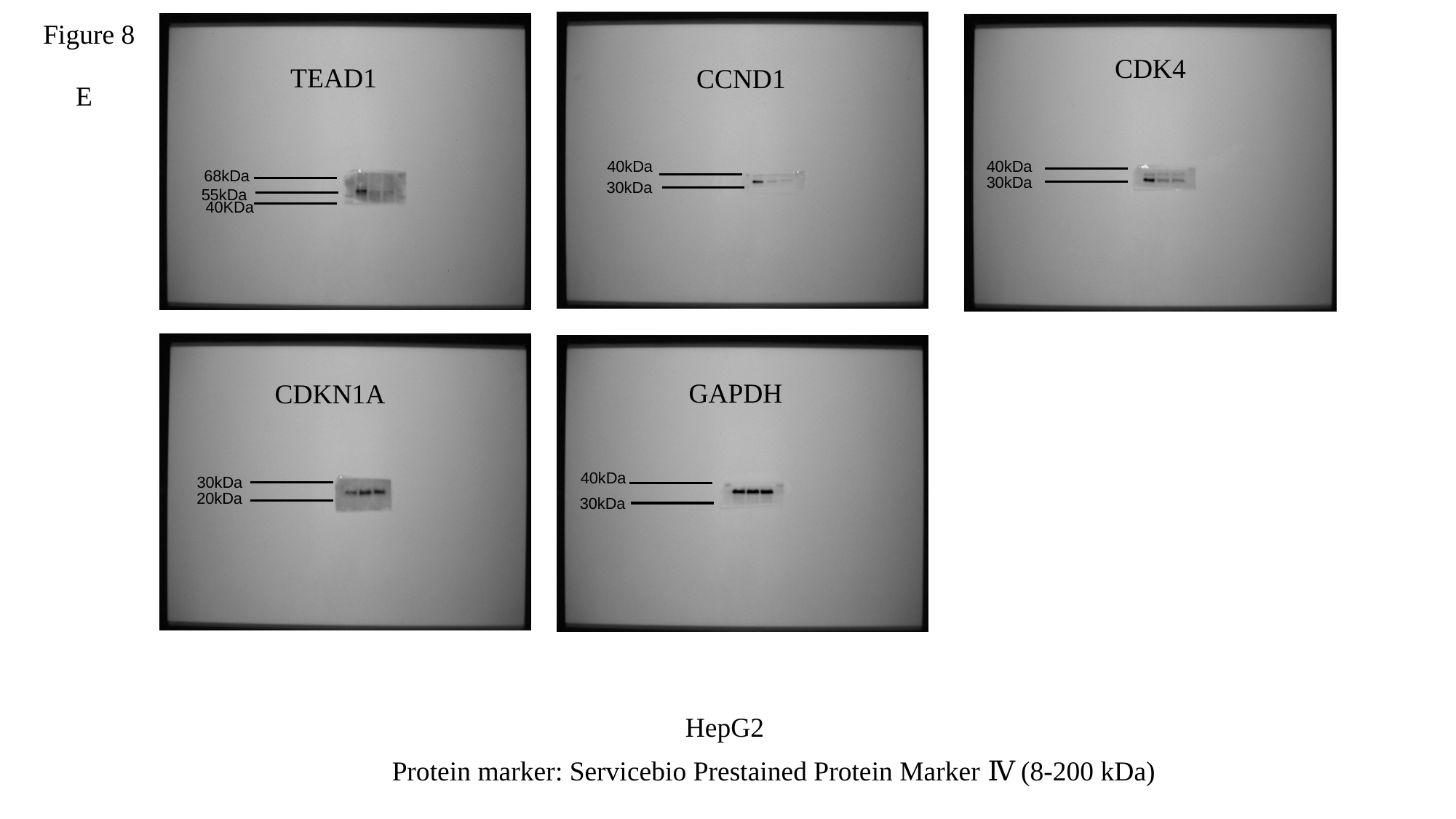

Figure 8
CDK4
TEAD1
CCND1
E
40kDa
40kDa
68kDa
30kDa
30kDa
55kDa
40KDa
GAPDH
CDKN1A
40kDa
30kDa
20kDa
30kDa
HepG2
Protein marker: Servicebio Prestained Protein Marker Ⅳ (8-200 kDa)

## Slide 3
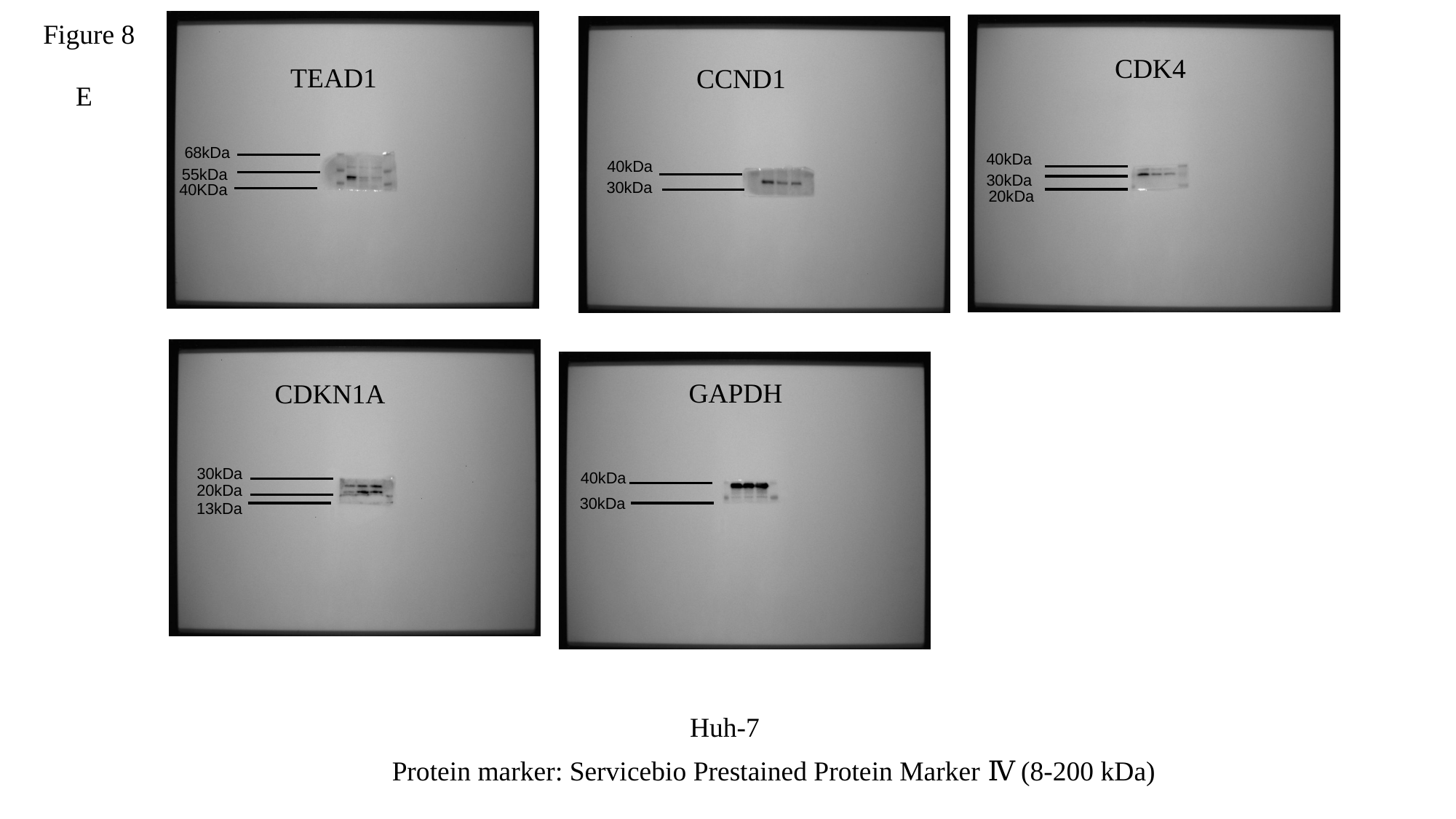

Figure 8
CDK4
TEAD1
CCND1
E
68kDa
40kDa
40kDa
55kDa
30kDa
30kDa
40KDa
20kDa
GAPDH
CDKN1A
30kDa
40kDa
20kDa
30kDa
13kDa
Huh-7
Protein marker: Servicebio Prestained Protein Marker Ⅳ (8-200 kDa)

## Slide 4
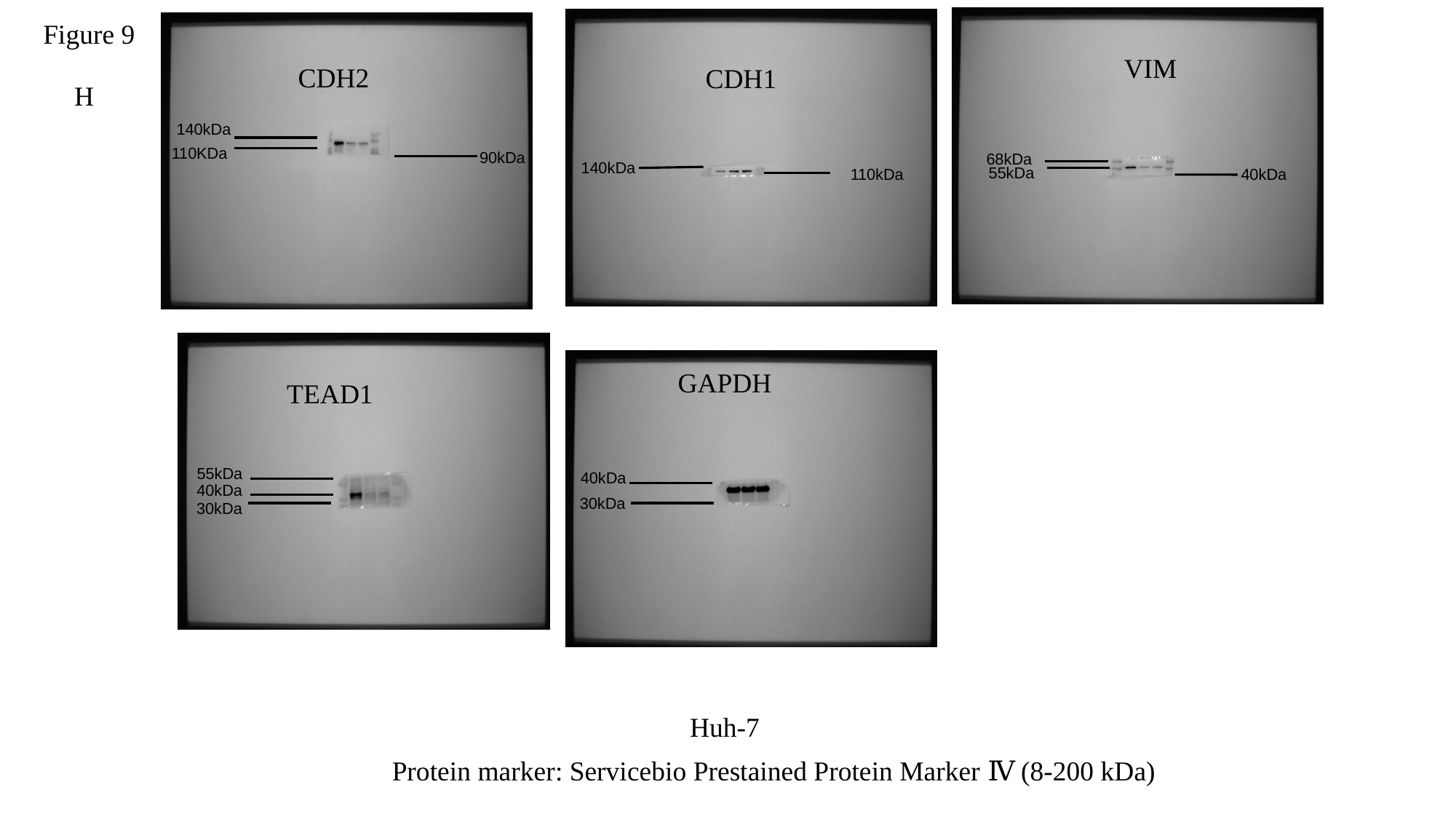

Figure 9
VIM
CDH2
CDH1
H
140kDa
110KDa
90kDa
68kDa
140kDa
55kDa
110kDa
40kDa
GAPDH
TEAD1
55kDa
40kDa
40kDa
30kDa
30kDa
Huh-7
Protein marker: Servicebio Prestained Protein Marker Ⅳ (8-200 kDa)

## Slide 5
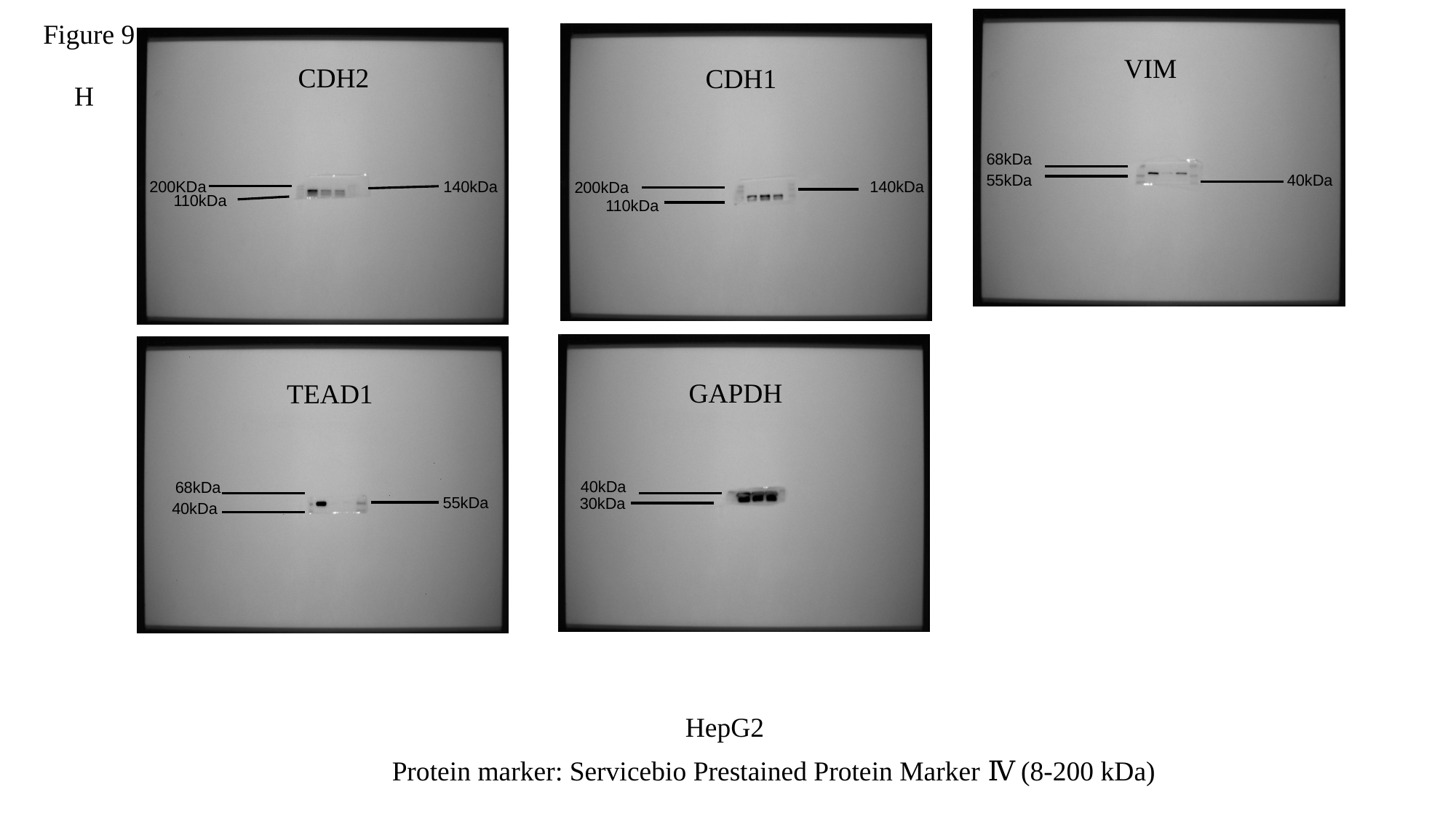

Figure 9
VIM
CDH2
CDH1
H
68kDa
55kDa
40kDa
140kDa
200KDa
140kDa
200kDa
110kDa
110kDa
GAPDH
TEAD1
40kDa
68kDa
55kDa
30kDa
40kDa
HepG2
Protein marker: Servicebio Prestained Protein Marker Ⅳ (8-200 kDa)
